# The CX3CR1/CX3CL1 (fractalkine) pathway in IgA vasculitis nephritis: guilty party or innocent bystander?

**DOI:** 10.1038/s41390-025-04385-3

**Published:** 2025-09-23

**Authors:** Patricio E. Ray, Matthew D. Griffin

**Affiliations:** 1 Child Health Research Center, University of Virginia School of Medicine, Charlottesville, VA USA; 2 Regenerative Medicine Institute (REMEDI) at CÚRAM Research Ireland Centre for Medical Devices, School of Medicine, University of Galway, Galway, Ireland

IgA vasculitis (IgAV) is a rare disease that occurs more commonly in children than in adults.^1^ Previously known as Henoch-Schönlein purpura, it presents with an acute, purpuric, non-blanching vasculitic rash which most frequently involves the lower extremities and is variously accompanied by joint, gastrointestinal, and renal involvement. When IgAV affects the kidneys, the condition is referred to as IgA vasculitis nephritis (IgAVN) and is identified clinically by micro- or macro-hematuria and proteinuria with or without hypertension and impaired renal function.^1^ Pathophysiologically, IgAV is characterized by small vessel inflammatory vasculitic lesions containing IgA1 dominant immune complexes in skin and other affected tissues. In the kidney, the glomerular and tubulointerstitial pathological abnormalities are indistinguishable from those of the more common glomerulonephritis, IgA nephropathy (IgAN), and, similar to IgAN, are associated with increased circulating levels of galactose-deficient IgA1 antibodies.^1,2^ Although IgAV is typically self-limiting in children, IgAVN is frequently treated with corticosteroids, sometimes combined with other immunosuppressants, in order to reduce inflammatory kidney injury. Even following initial response to therapy, however, a substantial proportion of children with biopsy-proven IgAVN eventually develop progressive decline in estimated glomerular filtration rate and are at risk for end-stage kidney disease.^1,2^ Thus, there is a significant need for new evidence generation to guide prognosis, personalized treatment, and long-term monitoring and management of children with IgAVN.^1^

In the current issue of *Pediatric Research*, Gu et al. provide new evidence that the immunological functions of the chemokine receptor CX3CR1 and its ligand CX3CL1 (also called fractalkine) may play an important role in the severity of intrarenal inflammation in IgAVN.^3^ The authors first performed quantitative analyses of the two components of the CX3CR1/fractalkine pathway in plasma and peripheral blood mononuclear cells (PBMC) collected from groups of children with newly-diagnosed IgAV without nephritis (*n* = 59), children with a clinical diagnosis of IgAVN (*n* = 42), and healthy control children (*n* = 26). These analyses indicated that plasma fractalkine concentrations were higher in children with non-nephritic IgAV than in healthy controls but were substantially higher again in those with IgAVN. Accompanying this finding, surface expression of the fractalkine receptor, CX3CR1, on circulating monocytes was also more frequent in children with IgAV compared to controls and in IgAVN compared to IgAV. Turning to the kidney, Gu et al. next performed immunohistochemical analyses of kidney biopsy tissue from 18 of the IgAVN cases, which were further subdivided into 11 with milder pathological grading and 9 with more severe pathology. Normal kidney tissue from nephrectomy samples of 8 children with renal tumors served as a control group. These studies revealed that staining intensities for fractalkine, CX3CR1, and the macrophage marker CD68 were sequentially higher in pathologically milder and more severe IgAVN compared to the control kidney. Plasma fractalkine and monocyte CX3CR1 expression were also higher in children with pathologically more severe compared to milder IgAVN. Correlative analyses between matched blood and kidney samples were generally negative for these indices, suggesting, perhaps, that the dynamics of the pathway activity differ in blood and tissue sites. Overarching conclusions from the study of Gu et al. would be that: (a) IgAV and IgAVN are associated with increased activity of the CX3CR1/fractalkine axis in the circulation. (b) Both circulating and intrarenal expression levels of this receptor/ligand pair are increased in children with IgAVN and appear to correlate with the pathological severity of IgAVN as well as with the density of macrophage infiltration.

These results provide evidence of association but not of causation. It is credible, however, that this immunological pathway could be of pathophysiological and clinical significance in IgAVN. CX3CR1 is expressed by key innate immune cells (monocytes, macrophages, dendritic cells, and natural killer (NK) cells) as well as by subpopulations of T cells in the circulation and within tissues.^4^ The binding of fractalkine to CX3CR1 represents an exclusive molecular partnership (i.e., neither is known to bind to other proteins) which mediates a range of important functional effects on immune cells. Because fractalkine is expressed as a cell surface-tethered protein but can also be released as a soluble ligand, these immunological effects occur at cell-cell interfaces as well as within blood and extracellular fluids.^4,5^ The expression and release of fractalkine and the consequences of its binding to CX3CR1 are predominantly associated with pro-inflammatory activity. Specifically, expression of fractalkine by endothelial, epithelial, and mesenchymal cells is induced in most tissues, including the kidney, by inflammatory stimuli and mediates chemotaxis, adhesion, maturation, and survival responses in CX3CR1^+^ immune cells.^4,5^ In their study, Gu et al. observed that intrarenal expression of fractalkine and CX3CR1 positively correlated with CD68, leading them to speculate that glomerular and interstitial macrophage infiltration is regulated by this pathway in IgAVN. In support of this, previous studies have shown that the fractalkine/CX3CR1 axis may play a role in the pathogenesis of IgAVN^6^ as well as in a range of acute and chronic kidney diseases, including IgAN and other vasculitides.^4,5^ Studies of subjects with IgAN have demonstrated increased plasma concentrations of fractalkine, which correlated with severity of renal inflammation and with renal survival,^7^ and have reported that glomerular and urinary fractalkine levels were associated with recurrent gross hematuria.^8^ In ANCA-associated vasculitis, increased glomerular endothelial expression of fractalkine has been reported in biopsies of patients with renal involvement, and anti-myeloperoxidase ANCA was shown to induce expression of fractalkine by glomerular endothelial cells, resulting in chemotaxis of inflammatory (CD16^+^) monocytes.^9^ Animal model studies of acute glomerulonephritis involving blockade or deficiency of CX3CR1/fractalkine binding have directly demonstrated the role of this pathway in mediating renal infiltration and adhesion of monocyte/macrophages.^5^

Although the results of Gu et al. are consistent with the broader literature implicating CX3CR1/fractalkine in the severity of IgAVN and other glomerular diseases, several caveats should be considered in interpreting the study. First, kidney expression of fractalkine, CX3CR1, and CD68 was assessed in a limited number of tissue samples either from children with IgAVN or from control children. The control samples were derived from normal regions adjacent to kidney tumors, and these children were significantly younger (median age ~3) than those in the IgAVN group (median age ~10 years). While the authors state that intrarenal fractalkine expression and numbers of CX3CR1^+^ and CD68^+^ cells are not affected by age, it is unclear whether this is supported by more extensive prior research. Second, the reported analyses were performed on samples taken at single time-points and the children were followed for limited periods. Thus, it remains unknown whether plasma fractalkine levels or proportions of CX3CR1^+^ cells changed in response to treatment or were predictive of short- or long-term clinical outcomes. Third, as no significant correlations were found between the plasma/blood cell analyses of CX3CR1/fractalkine and the kidney tissue-specific indices in children with biopsy-proven IgAVN, it seems unlikely that a blood-based assay of this pathway will become a clinically useful prognostic biomarker. In this regard, analysis of urinary fractalkine would have been of interest as a non-invasive approach to quantifying intrarenal activity of the pathway in children with IgAV and IgAVN.

While a putative role for increased activity of CX3CR1/fractalkine on monocyte/macrophage-mediated inflammation is of clear interest, previous studies have also suggested that fractalkine has important effects on cytotoxic T cells and NK cells in childhood IgAVN.^6^ The study by Gu et al. provides less insight into the potential role of fractalkine in regulating lymphocyte activation, trafficking, and effector functions. The authors found no significant differences between mild and severe cases in the proportions of CX3CR1^+^ lymphocytes in PBMC, but did not perform more discriminatory flow cytometry analyses of CD4 and CD8 T cells, NK cells, and B cells, and did not quantify or localize lymphocyte subtypes within kidney tissue samples. It should also be noted that, in some experimental settings, blockade or deficiency of CX3CR1 has been reported to be associated with exacerbation of kidney injury.^4,5^ Thus, more studies are needed to determine the full range of potential actions of the CX3CR1/fractalkine axis in the pathogenesis and outcome of childhood IgAVN, and to determine whether it could become a specific therapeutic target for children with more severe or progressive renal injury due to this disease.

Despite the limitations in scope that are understandably common in studies of rare childhood diseases, the study by Gu et al. represents a valuable new contribution, and we anticipate that it will stimulate further research into the pathogenesis of childhood vasculitis. Studies such as this are needed to better inform strategies for treating and monitoring IgAVN. Centers with a track record of enrolling cohorts of newly-diagnosed children with IgAV/IgAVN will also be essential for investigating emerging new therapies, including those currently under investigation to block CX3CR1/fractalkine or pathways (e.g., interleukin 6) that regulate other steps in the disease pathogenesis, such as the production of IgA1 from B cells. It is unclear, however, whether inhibiting one pro-inflammatory pathway will be sufficient to prevent renal injury caused by the trans-endothelial migration of leukocytes in IgAVN and other immune-mediated kidney diseases. Given the complexities of the regulation of inflammatory cell transmigration, differentiation and activation within the kidney, we wonder whether future therapies directed toward intrarenal immune cell infiltration may have to target multiple mediators—for example, by preventing the simultaneous binding of several chemokines (CCL2, CCL4, CCL5), cytokines (TNF, IL-1β, IFNγ) and growth factors (FGF-2, VEGF-A) to renal heparan sulfate proteoglycans.^10^
